# Native plant species growing on the abandoned Zaida lead/zinc mine site in Morocco: Phytoremediation potential for biomonitoring perspective

**DOI:** 10.1371/journal.pone.0305053

**Published:** 2024-06-26

**Authors:** Alassane Diallo, Said El Hasnaoui, Youssef Dallahi, Abdelaziz Smouni, Mouna Fahr

**Affiliations:** 1 Faculté des Sciences, Laboratoire de Biotechnologie et Physiologie Végétales, Centre de Biotechnologie Végétale et Microbienne Biodiversité et Environnement, Université Mohammed V de Rabat, Rabat, Morocco; 2 Laboratoire International Associé « Sciences, Environnements, Sociétés et Activités Minières » « LIA-SESAM », Université Mohammed V Morocco/ Université Laval, Laval, Canada; 3 Laboratoire Mixte International Activité Minière Responsable “LMI-AMIR”, IRD/UM5/INAU, Rabat, Morocco; 4 Centre d’Excellence Africain Mines et Environnement Minier, Institut National Polytechnique Félix HOUPHOUET BOIGNY, Yamoussoukro, Côte d’Ivoire; Universiti Brunei Darussalam, BRUNEI DARUSSALAM

## Abstract

This study aims to assess the level of metal contamination and the ecological risk index at the abandoned Zaida Pb/Zn mining site in eastern Morocco and identify native plant species found on the site that can be used in site rehabilitation through phytoremediation strategies. Samples from seven native and abundant plant species at the site, along with their rhizospheric soils, were collected and analyzed using Inductively Coupled Plasma Mass Spectrometry (ICP-MS) to determine the concentrations of various metal(loid)s, including As, Cu, Ni, Cd, Sb, Zn, and Pb. Indicators of soil pollution and ecological risks were also assessed, including the enrichment factor (EF), pollution index (PI), and ecological risk index (ERI). The Biological Accumulation Coefficient (BAC), Translocation Factor (TF), and Biological Concentration Factor (BCF) of plant samples were calculated. The results reveal polymetallic soil contamination, with notably higher concentrations of Pb, Cu and Zn, reaching respectively 5568 mg kg−1 DW, 152 mg kg−^1^ DW, and 148 mg kg−1 DW, indicating a significant potential ecological risk. The enrichment factor (EF) was also assessed for each metal(loid)s, and the results indicated that the metal contamination was of anthropogenic origin and linked to intensive mining activities in Zaida. These findings are supported by the pollution index (PI) ranging from 1.6 to 10.01, which reveals an extremely high metal(loid)s pollution level. None of the plant species exhibited a hyperaccumulation of metal(loid)s. However, *Artemisia herba alba* demonstrated a strong capacity to accumulate Pb in its aboveground parts, with a concentration of 468 mg kg−^1^ DW. *Stipa tenacissima*, *Retama spherocarpa*, and *Astragalus armatus*, showed a significant Pb accumulation in their roots reaching 280, 260, and 256 mg kg−^1^ DW.respectively. Based on BAC, TF, and BCF, *Stipa tenacissima* exhibited potential for Ni and Cd phytostabilization, as well as the ability for Zn phytoextraction. Additionally, *Artemisia herba alba* displayed the capability to phytoextract Cd and had a high propensity to translocate all the studied metal(loid)s. *Astragalus armatus* has the potential to be used in the phytostabilization of Zn and Ni, as well as for the phytoextraction of As and Sb. These native species from the Zaida site, although not hyperaccumulators, have the potential to contribute significantly to the phytoextraction or phytostabilization of potentially toxic elements (PTEs). Moreover, they can serve as vegetative cover to mitigate the erosion and dispersion of metal(loid)s.

## 1. Introduction

Mining operations contribute substantially to the economy by furnishing essential raw materials and energy to human society [1]. However, intensive mining activities generate a significant amount of contaminants during extraction, which are further dispersed during other processes, such as treatment and transport [2]. Among the various pollutants, Potentially Toxic Elements (PTEs) pose the greatest threat because of their significant environmental consequences and their ability to endure for an extended period [3] persisting even after the closure of mining operations [4]. The presence of these metal(loid) contaminants adversely affects human health as they enter the food chain [5]. Several studies have highlighted the toxicity of PTEs that are potentially dangerous to human health and can cause cancers, hematological and cardiovascular disorders, diabetes, hypoglycemia, and renal diseases [6–9]. Additionally, they contribute to the deterioration of both terrestrial and aquatic ecosystems as well as the loss of microbial diversity [10]. Research has identified over 5 million sites worldwide contaminated by heavy metals and metalloids, generating a global economic cost exceeding 10 billion US dollars annually [9]. In the Moroccan context, over 200 abandoned mining sites, accompanied by tons of contaminated waste left without treatment or rehabilitation, pose serious environmental problems [11]. Several techniques are available to remove metal(loid) contamination, including physicochemical methods[12]. Phytoremediation is the most eco-friendly and least costly method [13]. This approach consists of using plants to remediate metals from the soil or to stabilize them by means of degradation or detoxification mechanisms [14]. These specialized plants, known as metallophyte plants, have special characteristics that allow them to grow and reproduce in soils contaminated with PTEs without suffering their toxic effects [15]. They use various remedial strategies, including phytoextraction, which consists of depolluting PTE-contaminated soils using hyperaccumulators [16], and phytostabilization, which involves immobilizing PTEs through plant root adsorption, absorption, complexation, and precipitation of metal(loid)s in the rhizosphere zone [13]. However, degraded mining environments have several limitations for normal plant growth due to edaphoclimatic constraints. In response, pioneering and native species that grow naturally and spontaneously in these environments and adapt to local conditions have captured the interest of researchers [17]. These species have the potential to serve as essential phytoresources for the phytoremediation of polluted areas [18,19]. Many studies have specifically targeted the identification of indigenous species growing in various contaminated areas, particularly mining sites. Midhat, Ouazzani [15] identified several metallophyte plants at various Pb/Zn mining sites in Morocco and demonstrated their potential for phytoremediation. Similar studies were conducted in Tunisia [9], China [1], and India [20]. These studies involve the identification of species and determination of their accumulation capacities using phytoremediation parameters such as Biological Accumulation Concentration (BAC), Biological Concentration Factor (BCF), and Translocation Factor (TF). These parameters play a crucial role in assessing potential plants for the phytoremediation of contaminated sites. The abandoned mining site of Zaida represents a serious environmental issue due to the piles of untreated mining waste. However, there is a lack of studies addressing the indigenous plants of these regions and their potential utilization in phytoremediation. The present study aims to screen and identify indigenous and native plant species at the Zaida mine site in eastern Morocco and assess their potential to accumulate PTEs, thereby identifying suitable candidate species for different phytoremediation strategies.

## 2. Materials and methods

### 2.1 Mining site description and sample collection

**Fig 1:**
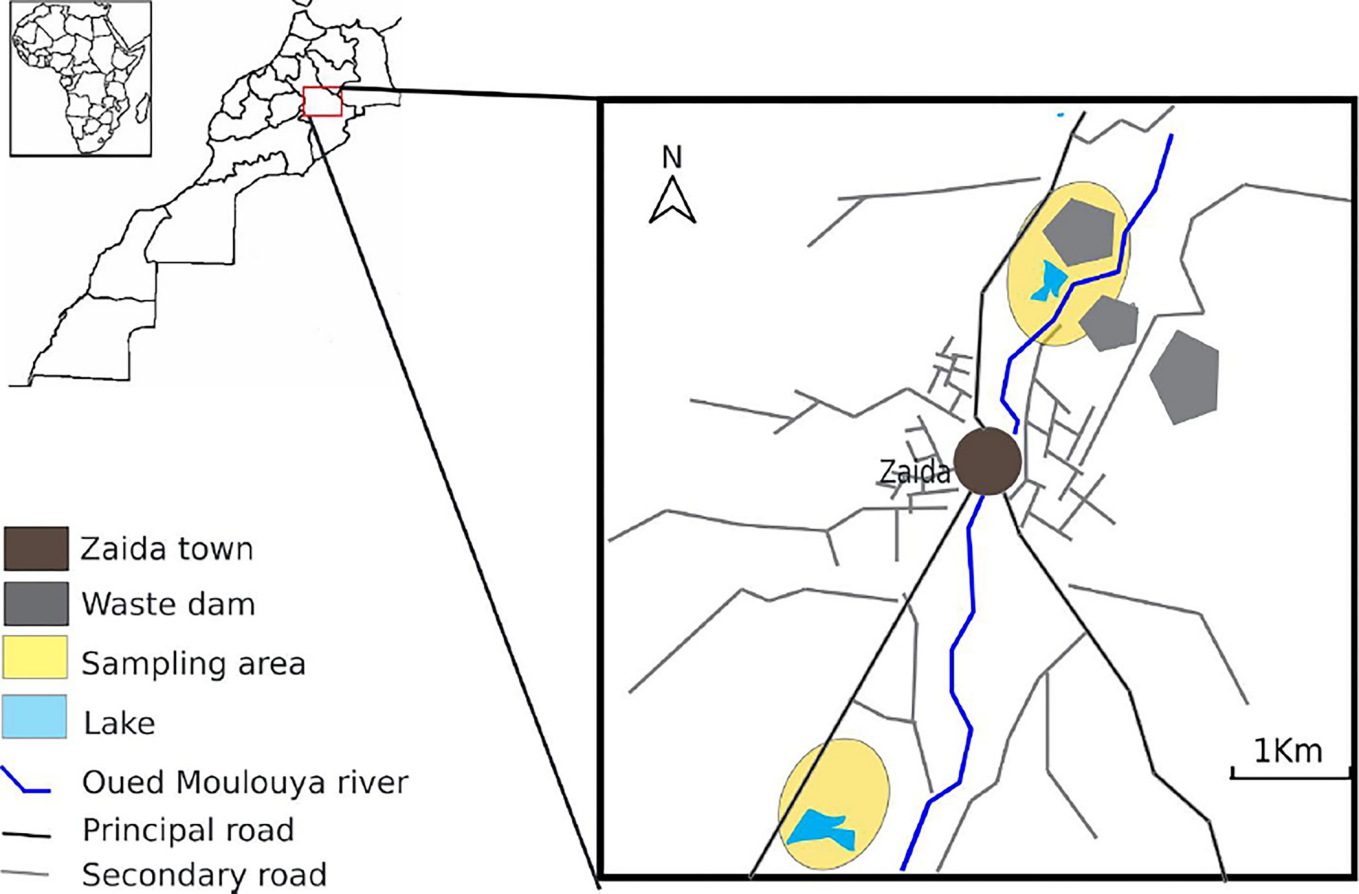
Plant and soil sampling area at Zaida abandoned mine.

The abandoned Zaida mine is located in the eastern region of Morocco at 32° 49’ 06" N, 4° 57’ 34" W, approximately 30 km northwest of the Midelt. It was the largest Pb mine in Morocco, producing approximately 630,172 tons of concentrated Pb (40–70% Pb) between 1972 and 1985 [21]. The mineralization consisted of 70% cerusite and 30% galena [22]. As mining activities ceased in 1985, mining waste produced during mining operations for a decade (around 70 Mt), was left in the open air without any remedial measures [21,23,24]. The region has a semi-arid climate that is affected by drought and disturbances due to mining activities [21].Soil and plant samples were collected from an abandoned Zaida mine in November 2022. A total of thirty-five samples of dominant plants were collected (7 species with 5 replicates per species). The plant species were identified according to the botanical flora references of Morocco [25]. Additionally, samples of rhizospheric soil were collected from depth of 0–20 cm (Fig 1).

### 2.2 Soil and plant analysis

The soil samples were carefully dried and ground to obtain fine particles (<2 mm) before use. Organic matter (OM) content was determined using the method described by Combs and Nathan [26]. Soil pH was determined on 5:1 water/soil suspension. The mixture was stirred for 1 hour using a magnetic stirrer and then decanted for 30 minutes. The pH was determined on the supernatant directly using a pH meter (“Bante210 Benchtop pH/mV Meter, Bante Instruments CO, LTD., Shanghai, China”) [23]. The total concentration of heavy metals was determined using the following steps: 50–100 mg of soil was weighed and placed into polytetrafluoroethylene (PTFE) tubes. 2 ml of concentrated nitric acid HNO_3_ (15N = 69%) of trace metal analysis (TMA) quality was added and the mixture is incubated overnight at room temperature. Then, 1.5 ml of concentrated HCl (12N = 37%) of TMA quality followed by 0.5 ml of HF 40% (28N) were added. The mixture is heated with the heating plates between 130°C and 150°C for five days, closing the PTFE tube plugs tightly. The mixture was cooled daily for 30 minutes before returning to heating. Five days later, after dissolving the soil sample, the mixture is quantitatively transferred to a 10 ml tube, and adjusting with distilled water.

The plant samples were separated into shoots and roots, and then thoroughly rinsed with flowing water to eliminate any residual substrate components. The samples underwent two 15-second rinses with a solution containing 0.2 mM CaSO4, followed by a final washing with distilled water. Plant samples were then dried at 72°C for 48 hours. Following this, they were further processed by being ground into a fine powder. The plant samples were digested according to the following protocol: 0.2 g of plant biomass was weighed and placed at the bottom of a PTFE tube. Then 1.5 ml of concentrated HNO_3_ (69%) was added. The mixture is incubated at room temperature for 6 hours. Subsequently, 1.5 ml of HCL (37%) was added and heated to 120°C on hot plates for approximately 6 hours. Then 1 ml of H_2_O_2_ concentrate (30% TMA) was added, and the mixture was reheated for 8 hours. Finally, after complete dissolution of the plant samples, the mixture was quantitatively transferred into a 10 ml tube and adjusted with distilled water.

Concentrations of metal(loid)s, including As, Cu, Ni, Cd, Sb, Zn, and Pb were determined in both soil and plant samples using ICP-MS (inductively coupled plasma-mass spectrometry; Ultima2 JY, Horiba Company, Edison, NJ, USA) at the CEREGE—European Center for Research and Teaching in Environmental Geoscience according to Marguí, Queralt [27], with the white values deduced from the measurement.

### 2.3 Assessment of soil pollution levels and phytoremediation factors

#### Soil Pollution Index (PI)

The soil pollution index is used to assess the level of heavy metal pollution. It is calculated by dividing metal concentrations in soil (mg kg^−1^) by the background metal concentration according to Chon, Ahn [28] and Wu, Teng [29]. The background concentration refers to the typical levels found in unpolluted soils according to Kabata-Pendias and Mukherjee [5]. Thus, the pollution index is calculated using the following equation:

PI=([As]soil/6+[Cd]soil/3+[Cu]soil/100+[Ni]soil/100+[Pb]soil/100+[Zn]soil/300+[Sb]soil/8.6)/7 (1)

[metal]soil: concentration in mg kg^−1^

The PI is categorized as low (PI ≤ 1), moderate (1 < PI ≤ 3), or high (PI > 3) according to Seklaoui, Boutaleb [30].

#### Soil Enrichment Factor (EF)

The enrichment factor assessment determines the origin of the contamination (natural or anthropogenic) [31]. The equation used for calculation is as follows, as stated by Bern, Walton-Day [32]:

EF=((Ci/Cref)sample∕(Ci/Cref)background) (2)

where (Ci/Cref) _sample_ is the ratio between the concentration of the relevant element and the reference element in the soil sample.

(Ci/Cref) _background_ is the ratio between the concentration of the element of interest and that of reference in the natural context (Average Crust). In this study, Scandium (Sc) was opted as a reference element considering its inanimate properties and having the least changes compared to the other samples [33]. Liu, Li [31] identified five contamination categories commonly acknowledged using the enrichment factor: EF < 1 indicates no enrichment, 1 ≤ EF < 10 suggests minimal enrichment, 10 ≤ EF < 100 indicates moderate enrichment, 100 ≤ EF < 1000 signifies significant enrichment, and EF > 1000 denotes extremely high enrichment.

#### Ecological Risk Index (ERI)

The ecological risk index, introduced by Hakanson in 1980, aims to assess the sensitivity of the biological community to a toxic substance and to estimate the potential ecological risk resulting from overall contamination [34]. This index takes into account the sensitivity of ecosystems, the concentration of metals, and their toxicity [35]. It involves on the sum of individual Ei values for each heavy metal when Ei represents the individual risk factor associated with heavy metal i (Eq 3) [36].


ERI=∑Ei (3)

In order to determine the ERI, the initial step involves calculating the accumulating coefficient Cf, which compares the concentration of a metallic element in a soil sample Ci to the reference concentration found in the average crust Cb (Eq 4).


Cf=Ci/Cb (4)

Then, calculate the potential ecological risk coefficient Ei, which is determined by multiplying the accumulation coefficient Cf by the toxic response factor Ti of a metallic element (Eq 5). According to Hakanson [35], Ti values for the PTEs studied are 10 for As, 5 for Cu, Ni, and Pb, 30 for Cd, and 1 for Zn.


Ei=Cf×Ti (5)

According to Ntakirutimana, Du [37], the potential ecological risk Ei is categorized into five classes: low (Ei < 40), moderate (40 ≤ Ei < 80), considerable (80 ≤ Ei < 160), high (160 ≤ Ei ≤ 320), and very high (Ei > 320).). The ERI was categorized into four classes: low (ERI <150), moderate (150 < ERI ≤ 300), high (300 < ERI ≤ 600), and very high (ERI > 600) [38].

#### Phytoremediation factors

To assess the level of accumulation of PTEs in different parts of different plant species, three factors were calculated:

- The biological concentration factor (BCF) is determined as the ratio of the metal content accumulated in the plant to the content present in the soil [39].

BCF=[Metal]root/[Metal]soil (6)

Plants that exhibit a BCF value greater than one are regarded as potential candidates suitable for phytostabilization [40].

- The translocation factor (TF) is the ability of a plant to transfer an element from its roots to its aerial part [41]. It is calculated using the following formula:

TF=[Metal]shoot/[Metal]root (7)

Plants with TF values greater than one have the potential for utilization in phytoextraction [41].

- Biological Accumulation Coefficient (BAC) was calculated as a ratio of heavy metal in shoots to that in soil [42]. It is calculated using the following formula:

BCF=[Metal]shoot/[Metal]soil (8)

## 3. Data analysis

Differences in metal concentrations within the roots and shoots of various species were examined through one-way ANOVA. This analysis was conducted separately for each metal in both the shoots and roots. Post hoc Tukey HSD tests were employed to pinpoint noteworthy pairwise distinctions between species concerning metal concentrations.Principal Component Analysis (PCA) was utilized to explore the relationships between metal concentrations in shoots and roots across the studied plant species. The data matrix encompasses the mean values (5 replicates) of each metal found in both the shoots and roots of each species. Both multivariate and univariate analyses were carried out using STATISTICA 10 (StatSoft Inc., Tulsa, OK, USA).

## 4. Results and discussion

### 4.1 Soil physicochemical characterization and potentially toxic elements concentrations at the mining sites

Metal(loid)s mobility and bioavailability are closely associated with the soil’s physicochemical properties [9]. The physicochemical parameters of soil samples collected from the abandoned mining site of Zaida were assessed. The pH ranged from 8.71 to 9.37, indicating that the soil in the Zaida mine is alkaline. This alkalinity can be attributed to the presence of carbonates [43]. Several studies have demonstrated the importance of soil pH in the solubility, availability, and precipitation of metals, as highlighted by Król, Mizerna [44]. Previous research has also established a correlation between the solubility and availability of metals. It has been observed that a low pH leads to greater mobility of metals in the soil, thereby reducing their availability, while a high pH decreases the mobility of metals such as Cd, Zn, Ni, Cu, and Pb [44,45]. The organic matter content (%OM), in the Zaida soil samples ranges from 0 to 5.36%. Laghlimi, Baghdad [43], conducted a study at the same site and found that the organic matter content ranges from 0.3 to 2.66%.

**Fig 2:**
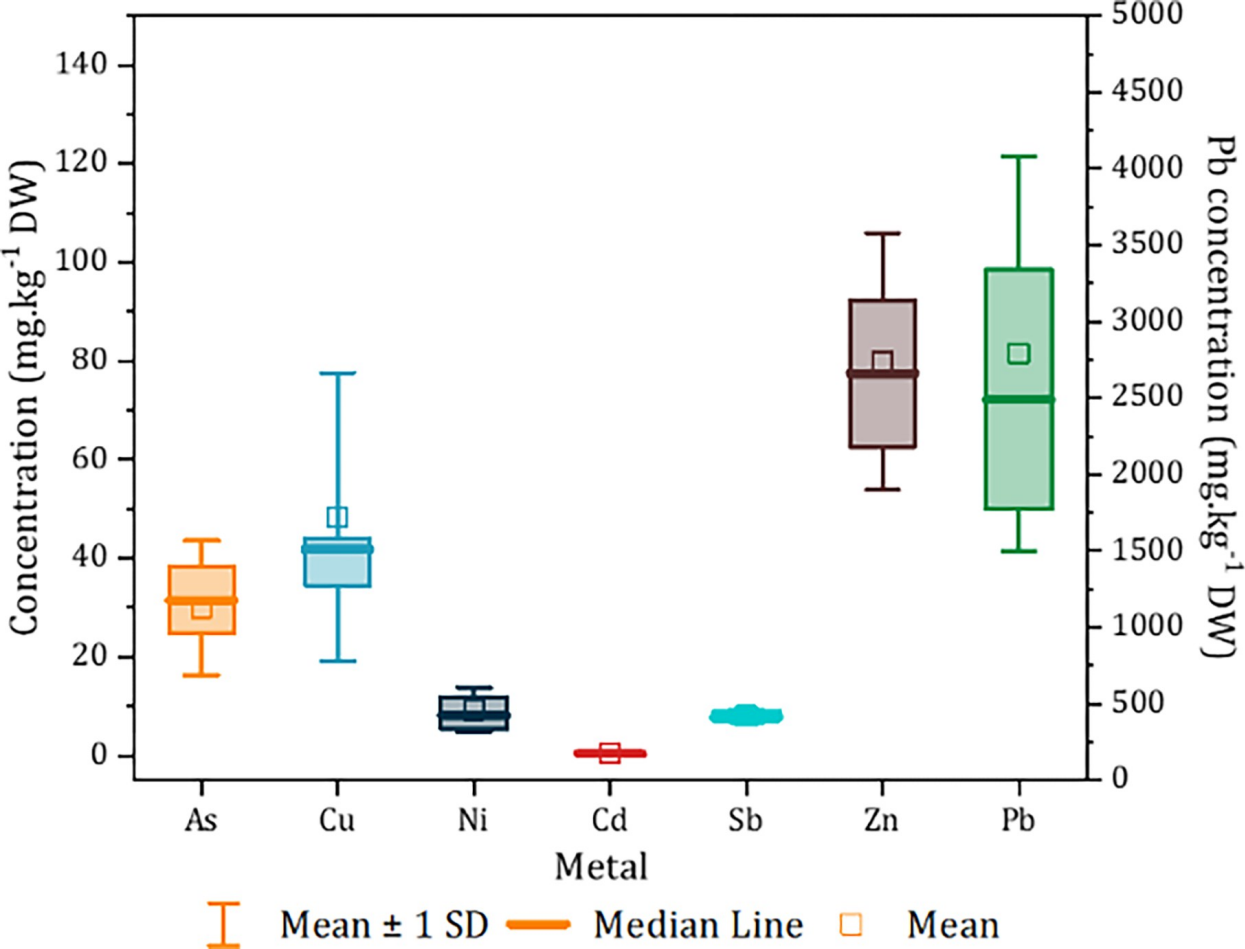
Metal(loid)s concentrations (mg kg^−1^ DW) in soil sampled from Zaida mine.

The levels of PTEs found in soil samples collected from the Zaida site are presented in Fig 2. According to the results in this figure, the concentrations of arsenic (As) range from 5.04 to 66.7 mg kg^−1^ DW, those of copper (Cu) from 27.7 to 152 mg kg^−1^ DW, nickel (Ni) from 4.86 to 22.7 mg kg^−1^ DW, cadmium (Cd) from 0.145 to 0.719 mg kg^−1^ DW, antimony (Sb) from 5.69 to 11 mg kg^−1^ DW, zinc (Zn) from 33.6 to 148 mg kg^−1^ DW, and lead (Pb) from 900 to 5568 mg kg^−1^. These concentrations exceed the soil reference values (background) set by Kabata-Pendias and Mukherjee [5], which are 6.8, 38.9, 29, 0.4, 0.7, 29, and 27 for As, Cu, Ni, Cd, Sb, Zn, and Pb, respectively. The metal concentrations analyzed were classified in the following order: Pb > Cu > Zn > As > Ni > Sb > Cd. The soil pollution index (PI) is used to assess the level of heavy metal pollution, and it ranges from 1.57 to 10.08 (Table 1). According to Seklaoui, Boutaleb [30], these values indicate that the site has extremely high heavy metal levels and is characterized by polymetallic contamination.

**Table 1:** The potential ecological risk coefficient (Ei) and the potential ecological risk index (ERI) of soil in Zaida abandoned mining site.

	As	Cu	Ni	Cd	Sb	Zn	Pb
Concentration in soils (mg kg^−1^)	29.9	48.4	9.28	0.414	8.09	79.9	2791
Average crust Cb [1]	6.8	38.9	29.0	0.4	0.7	29.0	27.0
Cf	4.40	1.24	0.320	1.03	11.6	2.76	103
Ti [2]	10.0	5.0	5.0	30.0	7.0	1.0	5.0
Ei	44.0	6.22	1.60	31.1	80.9	2.76	517
ERI	683						

[1]: Background crust according by Kabata-Pendias and Mukherjee [5].

[2]: Toxic response factor Ti according by Hakanson [35].

**Fig 3:**
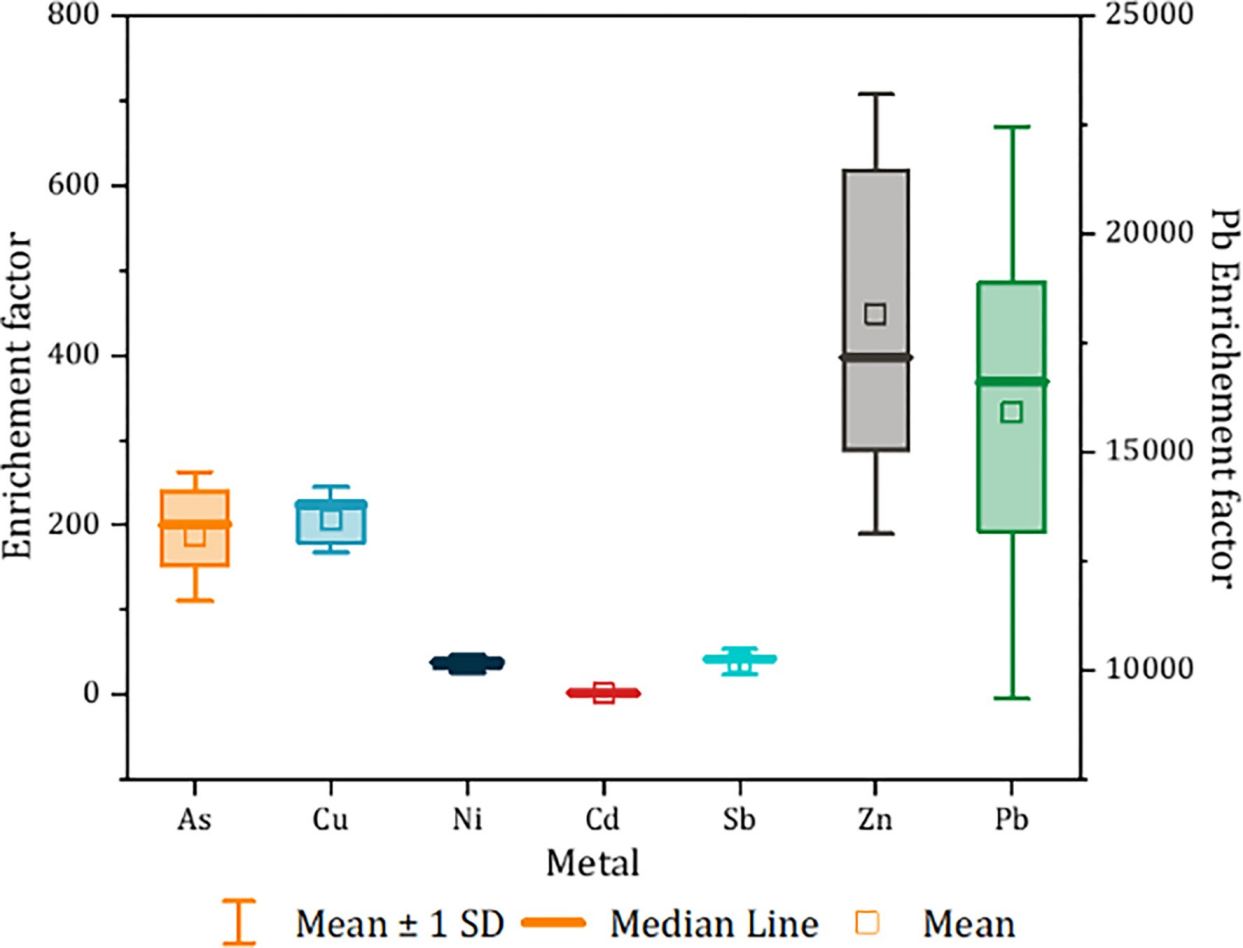
Metal Enrichment Factor in soils sampled from the Zaida mine.

The enrichment factor (EF) defines the concentrations of a given element at a specific location relative to the average natural occurrences, enabling the identification of potential pollution sources [31,46]. As, Cu, Ni, Cd, Sb, Zn, and Pb have computed EF values of 269, 241, 49.4, 2.993, 54.4, 864, and 24897, respectively (Fig 3). These enrichments of the metal(loids), notably Pb and Zn, can be attributed to the mining activities at the site. According to Liu, Li [31], this contamination is a result of anthropogenic activities (human activities). Several research studies have investigated the characterization of metal contamination in abandoned mining sites in Morocco. Laghlimi, Baghdad [43] conducted a comparable investigation of the soil from the abandoned mines in Zaida. Overall, Zn concentrations ranged from 48.50 mg kg^-1^ DW to 830 mg kg^-1^ DW, Pb values ranged from 0.36 to 830 mg kg^-1^ DW, Cu values varied from 0.11 to 77.2 mg kg^-1^ DW, and Cd concentrations ranged from 0.03 to 3 mg kg^-1^ DW. The current study’s findings suggest a lower level of contamination, potentially due to analytical methods and pedoclimatic conditions. El Hasnaoui, Fahr [18] found soil heavy metal (HM) concentrations similar to our findings at a Pb and Zn mining site in Touissit, Morocco. In their study, HM concentrations ranged from 43 to 82.90 mg kg^−1^ DW for As, from15 to 36 mg kg^−1^ DW for Cd, from 328 to1405 mg kg^−1^ DW, from 9 to 16 mg kg^−1^ DW for Ni, from 6445 to 18.324 mg kg^−1^ DW for Pb, from 96.71 to 242.80 mg kg^−1^ DW for Sb, and from 2096 to 5385 mg kg^−1^ for Zn (El Hasnaoui, Fahr [18].

Despite this significant polymetallic contamination with a high ecological risk, several plant species grow normally in this site and have developed capacities for heavy metal tolerance. Consequently, they can be used for phytoremediation purposes at polluted sites.

### 4.2 Ecological risk index assessment

The ecological risk coefficient (Ei) was calculated according to Hakanson [35], and the obtained results indicate the following order, Pb > Sb > As > Cd > Cu > Zn > Ni (Table 1). The calculated values for As, Cu, Ni, Cd, Sb, Zn, and Pb are 44, 6.22, 1.60, 31.1, 80.9, 2.76, and 517, respectively. According to Ntakirutimana, Du [37], Cu, Ni, Cd, and Zn exhibit a low ecological risk with Ei values less than 40. As falls within the moderate Ei category (40 ≤ Ei < 80). Conversely, Sb demonstrates an Ei slightly exceeding 80, indicating a significant risk potential (80 ≤ Ei < 160). However, Pb displays a notably high Ei value (516.9517), signifying a high ecological risk. On the same abandoned mining site, El Azhari, Rhoujjati [47] reported a high potential ecological risk associated with Pb, indicating a significant and severe ecological risk. The study’s findings indicate that the Zaida tailings not only represent a possible source of contamination but also constitute a serious threat to the local ecological systems [47]. Arab, Boutaleb [38], reported similar at abandoned polymetallic site in the Kef Oum Teboul district. The ERI was also calculated following Liu, Zhuo [36], which involved the sum of the Ei values for all the metal(loid)s analyzed (Table 1). The total Ei value for As, Cu, Ni, Cd, Sb, Zn, and Pb is 683. According to the ranking established by Ntakirutimana, Du [37], the ERI in this study is classified as very high, falling within the range of ERI > 600. Other studies were carried out on a Pb-Zn mining site in Changhua, demonstrating a polymetallic contamination of Cu, Pb, Zn, and Cd, which resulted in a severe ecological risk index [34]. Baghaie and Aghili [48], conducted a study on a Pb-Zn mine in the central part of Iran, specifically in Nakhlak, revealing an Ecological Risk Index (ERI) ranging from 150 to 300, indicating a moderate ecological risk.

### 4.3 Potentially toxic elements concentrations in plants

Seven dominant plant species belonging to five distinct families were identified and collected at the Zaida abandoned mining site (Table 2). Among them, *Retama spherocarpa* and *Astragalus armatus* belong to the Fabaceae family; *Noaea mucronata* and *Salsola vermiculata* fall under the Amaranthaceae family; and *Artemisia herba alba*, *Stipa tenacissima*, and *Peganum harmala* are classified within the Asteraceae, Poaceae, and Nitrariaceae families, respectively.

**Table 2:** List of dominant plant species in the vicinity of the studied site.

**Plant species**	**Family**
*Retama spherocarpa*	*Fabaceae*
*Astragalus armatus*	
*Noaea mucronata*	*Amaranthaceae*
*Salsola vermiculata*	
*Artemisia herba alba*	*Asteraceae*
*Stipa tenacissima*	*Poaceae*
*Peganum harmala*	*Nitrariaceae*

Metal concentrations in plant shoots and roots were assayed (Tables 3 and 4). The distribution and accumulation of metal(loid)s in the different plant tissues depend on both the level of metal pollution and the plant species [15,49]. In the plant species examined, there were notable and statistically significant variations in metal concentrations, as indicated by a p-value (P < 0.05) (Table 3).

**Table 3:** Results of variance analysis (One-way ANOVA) comparing the difference in metal concentration among species (dF = 6).

	Shoots		Roots	
** **	*F*	*P*	*F*	*P*
**As**	9.51	<0.0001	21.7	<0.0001
**Cu**	18.7	<0.0001	8.49	0.000134
**Ni**	25.6	<0.0001	28.0	<0.0001
**Cd**	6.67	0.000725	6.45	0.001154
**Sb**	9.81	0.000257	12.5	<0.0001
**Zn**	17.7	<0.0001	20.3	<0.0001
**Pb**	58.5	<0.0001	26.1	<0.0001

**Table 4:** Potentially toxic elements concentrations in shoots and roots (data in mg kg^−1^ DW) of dominant plant species collected from the studied site.

Species	Part	As	Cu	Ni	Cd	Sb	Zn	Pb
*Retama spherocarpa*	Shoot	0.462 ± 0.19 ^**ab**^	5.68 ± 1.27 ^**a**^	4.63 ± 0.47 ^**bcd**^	0.190 ± 0.08 ^**ab**^	0.099 ± 0.01 ^**ab**^	73.3 ± 19.05 ^**ab**^	47.4 ± 15.09 ^**a**^
	Root	1.16 ± 0.15 ^**ab**^	7.77 ± 2.83 ^**ab**^	4.79 ± 0.85 ^**bcd**^	0.372 ± 0.14 ^**abcde**^	0.201 ± 0.09 ^**ab**^	106 ± 66.55 ^**b**^	260 ± 44.34 ^**c**^
*Stipa tenacissima*	Shoot	1.50 ± 0.45 ^**b**^	11.9 ± 0.46 ^**abc**^	7.02 ± 1.62 ^**d**^	0.515 ± 0.16 ^**cde**^	0.468 ± 0.13 ^**ab**^	48.2 ± 6.74 ^**ab**^	249 ± 14.86 ^**bc**^
	Root	1.55 ± 0.22 ^**ab**^	14 ± 3.11 ^**bcde**^	12.3 ± 2.79 ^**e**^	0.570 ± 0.06 ^**de**^	0.551 ± 0.16 ^**bc**^	32.1 ± 3.63 ^**a**^	280 ± 72.35 ^**c**^
*Artemisia herba alba*	Shoot	3.25 ± 1.33 ^**c**^	19.3 ± 4.28 ^**d**^	6.55 ± 1.33 ^**d**^	0.575 ± 0.15 ^**e**^	0.957 ± 0.37 ^**cd**^	46.4 ± 13.76 ^**ab**^	468 ± 99.34 ^**d**^
	Root	0.590 ± 0.36 ^**ab**^	17.8 ± 7 ^**de**^	5.60 ± 0.75 ^**cd**^	0.481 ± 0.24 ^**bcde**^	0.354 ± 0.17 ^**ab**^	27.2 ± 8.12 ^**a**^	77.6 ± 31.48 ^**a**^
*Astragalus armatus*	Shoot	1.23 ± 0.58 ^**ab**^	12.6 ± 0.86 ^**abcde**^	6.93 ± 0.79 ^**d**^	0.244 ± 0.08 ^**abc**^	1.15 ± 0.54 ^**d**^	69.0 ± 14.42 ^**ab**^	253 ± 28.3 ^**bc**^
	Root	0.570 ± 0.26 ^**ab**^	16.7 ± 3.84 ^**cde**^	7.03 ± 1.75 ^**d**^	0.273 ± 0.08 ^**abcd**^	0.265 ± 0.02 ^**ab**^	263 ± 80.54 ^**c**^	256 ± 69.24 ^**bc**^
*Salsola vermiculata*	Shoot	1.03 ± 0.63 ^**ab**^	6.84 ± 1.91 ^**a**^	2.98 ± 0.64 ^**abc**^	0.286 ± 0.15 ^**abcd**^	0.166 ± 0.03 ^**ab**^	16.9 ± 6.43 ^**a**^	98.8 ± 10.88 ^**a**^
	Root	0.367 ± 0.06 ^**ab**^	6.87 ± 1.63 ^**a**^	2.72 ± 0.7 ^**ab**^	0.153 ± 0.02 ^**a**^	0.122 ± 0.02 ^**ab**^	14.1 ± 2.89 ^**a**^	52.7 ± 16 ^**a**^
*Noaea mucronata*	Shoot	0.362 ± 0.09 ^**ab**^	7.41 ± 0.88 ^**ab**^	3 ± 0.26 ^**abc**^	0.370 ± 0.09 ^**abcde**^	0.163 ± 0.08 ^**ab**^	12.9 ± 3.77 ^**a**^	17.3 ± 6.06 ^**a**^
	Root	0.274 ± 0.05 ^**a**^	6.63 ± 1.45 ^**a**^	2.56 ± 0.4 ^**ab**^	0.170 ± 0.03 ^**abc**^	0.069 ± 0.02 ^**a**^	8.69 ± 2.52 ^**a**^	13.1 ± 3.81 ^**a**^
*Peganum harmala*	Shoot	1.09 ± 0.39 ^**ab**^	12.6 ± 2.82 ^**abce**^	1.45 ± 0.49 ^**a**^	0.141 ± 0.09	ND	33.2 ± 5.25 ^**a**^	13.8 ± 8.14 ^**a**^
	Root	ND	ND	ND	ND	ND	17.4 ± 2.99 ^**a**^	28.*6* ± 9.69 ^**a**^

ND: Not determined.

Explanation: The values in the table are the average of heavy metal concentrations in both roots and shoots for each plant species. Averages followed by the same letters indicate no significant differences between them.

#### a. Arsenic (As)

The arsenic (As) levels in all samples remain below the toxicity threshold set by Krämer [50] and Kabata-Pendias and Mukherjee [5], which defined as above 80 mg kg^−1^ DW. Except for *Retama spherocarpa* and *Stipa tenacissima*, all other species exhibit higher concentrations of As in their shoots compared to the root levels. *Artemisia herba alba* has values of 3.25 mg kg^−1^ DW in shoots versus 0.590 mg kg^−1^ DW in roots, 1.23 and 0.570 mg kg^−1^ DW for *Astragalus armatus*, 1.03 and 0.367 mg kg^−1^ DW for *Salsola vermiculata*, and 0.362 and 0.274 mg kg^−1^ DW for *Noaea micronata* (Table 4) in shoots and roots, respectively. This low accumulation of As is attributed to its low mobility within the plant, a trait commonly observed except in hyperaccumulator plants [51]. None of the species reached the hyperaccumulation threshold of As, which is > 1000 mg kg^−1^ DW according to Ghaderian, Lyon [52]. Cruzado-Tafur, Bierla [53] also reported low As accumulation in the aboveground parts in several species collected from the Ag–Pb/Zn site in the Hualgayoc district (northern Peru), such as *Hypericum laricifolium* (2.01 mg kg^−1^), *Achyrocline alata* (3.82 mg kg^−1^), *Arenaria digyna* (3.93 mg kg^−1^), *Calamagrostis recta* (3.99 mg kg^−1^), *Puya* sp. (5.12 mg kg^−1^), *Ageratina glechonophylla* (6.44 mg kg^−1^), and *Chusquea scandens* (4.76 mg kg^−1^). On an abandoned Pb-Ag-Zn mining site located in the Mazarron district (southeastern Spain) Azizi, Faz [54] found that the species *A*. *herba-alba* accumulated up to 7.144 mg kg^−1^ for As. In contrast to our findings, Hosseini, Rezazadeh [55] observed a significant accumulation of As (19.3 to 5430 mg kg^−1^) in the roots of *D*. *glomerata*, a species belonging to the Poaceae family, collected from an iron mine in northwest Iran.

Biological concentration factors (BCF), translocation factors (TF), and biological accumulation coefficients (BAC) (Table 5) are used to assess the plants’ ability to accumulate metal(loid)s. If both BAC and TF values are higher than 1, the plants are regarded as candidates for phytoextraction. However, if BCF is higher than 1 while TF is lower than 1, these plants are considered candidates for phytostabilization [56,57].

**Table 5:** Bioconcentration factor (BCF), Translocation factor (TF) and Biological accumulation coefficient (BAC) for the studied plants species.

Species	Factor	As	Cu	Ni	Cd	Sb	Zn	Pb
***Retama spherocarpa***	BAC	0.014	0.133	0.642	0.363	0.011	0.661	0.011
	TF	0.398	0.732	0.967	0.513	0.491	0.689	0.182
	BCF	0.035	0.182	0.663	0.707	0.022	0.960	0.061
***Stipa tenacissima***	BAC	0.042	0.310	**1.32**	**1.14**	**0.057**	**0.663**	**0.116**
	TF	0.966	0.853	**0.571**	**0.904**	**0.850**	**1.50**	**0.890**
	BCF	0.044	0.363	**2.31**	**1.27**	**0.067**	**0.442**	**0.130**
***Artemisia herba alba***	BAC	0.110	0.468	0.784	**1.38**	0.123	0.578	0.146
	TF	**5.52**	**1.08**	**1.17**	**1.20**	**2.70**	**1.71**	**6.04**
	BCF	0.020	0.432	0.669	**1.15**	0.046	0.338	0.024
***Astragalus armatus***	BAC	0.042	0.302	**1.12**	0.596	0.159	0.593	0.076
	TF	**2.17**	0.750	0.986	0.893	**4.35**	0.263	0.991
	BCF	0.019	0.403	**1.13**	0.668	0.037	**2.26**	0.076
***Salsola vermiculata***	BAC	0.032	0.188	0.456	0.735	0.021	0.240	0.036
	TF	**2.81**	0.995	**1.09**	**1.87**	**1.35**	**1.20**	**1.87**
	BCF	0.012	0.189	0.417	0.392	0.016	0.201	0.019
***Noaea mucronata***	BAC	0.007	0.056	0.172	0.812	0.020	0.148	0.009
	TF	**1.32**	**1.12**	**1.17**	**2.18**	**2.34**	**1.48**	**1.32**
	BCF	0.006	0.050	0.147	0.373	0.008	0.100	0.007
***Peganum harmala***	BAC	0.185	0.363	0.118	0.566	ND	0.776	0.010
	TF	ND	ND	ND	ND	ND	**1.91**	0.482
	BCF	ND	ND	ND	ND	ND	0.407	0.021

ND: Not determined.

**Bold values** indicate BCF, TF and BAC values greater than 1.

For As, none of the examined species exhibited a BCF or BAC exceeding 1, suggesting that they do not accumulate substantial amounts of As in either their aboveground or root parts. However, with the exception of *Retama spherocarpa* and *Stipa tenacissima*, which exhibited a TF below 1 (0.398 and 0.966, respectively), the other species demonstrated TF values of 5.52, 2.17, 2.81 and 1.32 for *Artemisia herba alba*, *Astragalus armatus*, *Salsola vermiculata*, and *Noaea mucronata*, respectively. This suggests that these species have the ability to translocate As to the aboveground parts. El Hasnaoui, Fahr [18] found that *Artemisia herba alba* grown in Pb and Zn mining site exhibited TF values of 1.04 for As. However, Azizi, Faz [54] reported that *Stipa tenacissima* and *Artemisia herba alba* accumulate high amounts of As in their roots (TF < 1), which means that, contrary to our results, this species could be used to reduce the migration of As in the soil. Hosseini, Rezazadeh [55] also observed low translocation of arsenic (TF < 1) in *D*. *glomerata*, harvested from an iron mine in northwest Iran. This limited translocation of As to aboveground parts represents a tolerance strategy in plants [58–60].

#### b. Copper (Cu)

The Cu concentrations found in the different species did not reach the toxicity threshold, which falls within the range of 20 to 30 according to Krämer [50] and Kabata-Pendias and Mukherjee [5]. Furthermore, there were no significant differences in the Cu concentrations between the aboveground and root parts within the same species (Table 4). However, three species accumulated slightly higher concentrations in their root parts compared to their aboveground parts, namely *Retama spherocarpa* (7.77 and 5.68 mg kg^−1^ DW), *Stipa tenacissima* (14 and 11.9 mg kg^−1^ DW), and *Astragalus armatus* (16.7 and 12.6 mg kg^−1^ DW). *Artemisia herba alba* and *Noaea mucronata* recorded slightly higher concentrations in their aboveground parts compared to their root parts. Midhat, Ouazzani [15] observed Cu concentrations ranging from 0.23 to 66.96 mg kg^−1^ in the aboveground parts and from 0.02 to 72.13 mg kg^−1^ in the roots of plants collected from three mining sites in the central-southern region of Morocco. Among these plants, *Forsskaolea tenacissima* L. and *Stipa capensis* Thunb., collected from the Pb-Zn and pyrite site at Sidi Bou-Othmane, recorded the highest Cu concentration in their aboveground parts (60.67 mg kg^−1^, and 56.72 mg kg^−1^ respectively). In a similar study conducted at two Pb/Zn sites in eastern Morocco, El Hasnaoui, Fahr [18] found that among 14 collected species, *S*. *tenacissima* and *A*. *herba-alba* exhibited significantly higher Cu concentrations in their root than other species (237.8 and 203.1 mg kg^−1^ dry matter, respectively). Cu is an essential micronutrient for plant nutrition and is involved in a range of plant growth and development processes (Midhat et al., 2019). The relatively low Cu values in the plant tissues could be attributed to limited Cu translocation, which prevents Cu toxicity and interference with essential processes, especially photosynthesis (Bech et al., 2017). None of the studied species is considered as hyperaccumulator plant for Cu. Cu hyperaccumulation requires a concentration higher than 1000 mg kg−^1^ DW in the aboveground part [61], a requirement considered difficult to achieve according to Van der Ent, Baker [62]. Indeed, only a specific group of plants, mainly found in Central Africa, are Cu hyperaccumulator plants [63].

None of the studied species exhibited a BCF or BAC value exceeding 1 for Cu (Table 5). This suggests that the studied species did not significantly accumulate high Cu concentrations in their aboveground and root parts. Midhat, Ouazzani [15] reported comparable findings in Pb and Zn mining sites situated in the southern center of Morocco, where none of the 46 indigenous plant species exhibited a BCF greater than 1. In the present study, two species showed a TF > 1 namely *Artemisia herba alba* (TF = 1.08), and *Noaea mucronata* (TF = 1.12). These species demonstrated the ability to translocate Cu from the roots to the aboveground parts, which is one of the criteria for phytoextraction. Siyar, Doulati Ardejani [56] found that *Artemisia herba alba* and *Stipa* sp, collected from a Cu smelter and refinery site in Khatunabad (Iran) recorded TF values for Cu of 1.02 and 1.84, respectively, thus demonstrating their ability for Cu phytoextraction. In an Ag-Pb/Zn mining site in southeastern Spain, Azizi, Faz [54] reported a BCF > 1 and TF < 1 for Cu in *Stipa tenacissima* and *Artemisia herba alba*, harvested in the vicinity of a Pb-(Ag)-Zn mine, which means they are suitable for Cu phytostabilization. These divergent results can be explained by the following factors: (i) metal accumulation in plants is determined by the total and available metal in the soil, (ii) environmental factors such as rainfall deposition and temperature had a substantial impact on metal uptake behavior; (iii) Finally, the source of HMs and their bioavailability influenced metal accumulation in plants considerably [64].

#### c. Nickel (Ni)

All the studied plant species showed low concentrations of Ni in their tissues, which did not exceed the toxicity threshold of 50 mg kg^−1^ DW defined by Krämer [50] (Table 4). Among them, *Stipa tenacissima* recorded the highest Ni concentration of 12.3 mg kg^−1^ DW in the root part, followed by *Astragalus armatus* with 7.03 mg kg^−1^ DW in the root part. *Retama spherocarpa*, *Artemisia herba alba*, *Salsola vermiculata*, and *Noaea mucronata* showed relatively lower concentrations (4.63 and 4.79 mg kg^−1^ DW, 6.55 and 5.60 mg kg^−1^ DW, 2.98 and 2.72 mg kg^−1^ DW, and 3 and 2.56 mg kg^−1^ DW, in their aboveground and root parts, respectively). Similar results were reported by Hosseini, Rezazadeh [55], showing that the concentrations of Ni in plant tissues collected from an iron site in northwest Iran ranged from 5.6 to 28.7 mg kg^−1^ in the roots and from 1.19 to 17.2 mg kg^−1^ in the aerial parts. None of the studied species reached the Ni hyperaccumulation threshold as defined by Reeves [65].

Only *Stipa tenacissima* and *Astragalus armatus* exhibited BAC and BCF values greater than one, indicating their ability to accumulate Ni in root parts (Table 5). This finding suggests that *Stipa tenacissima* and *Astragalus armatus* have a good potential for Ni phytostabilization. Infact, *Artemisia herba alba*, *Salsola vermiculata*, and *Noaea mucronata* recorded TF values greater than 1 (1.17, 1.09, and 1.17, respectively), indicating a higher tendency to translocate and accumulate Ni in their aboveground parts. A study conducted by Siyar, Doulati Ardejani [56] at a Cu smelter and refinery in Iran reveals that *Artemisia* sp. and *Peganum harmala* recorded a TF greater than 1 for Ni, suggesting their ability to translocate this metal. Similarly, El Hasnaoui, Fahr [18] reported a TF greater than 1 in 8 species collected from two Pb/Zn sites in eastern Morocco namely *C*. *libanotis* (1.27), *A*. *herba-alba* (1.24), *C*. *bursa-pastoris* (collected from Oued el Heimer site) (1.24), *P*. *communis* (1.17), *C*. *althaeoides* (1.17), *S*. *hispanicus* L. (1.15), *C*. *bursa-pastoris* (collected from Touissite site) (1.08), and *A*. *alopecuroides* (1.05).

#### d. Cadmium (Cd)

Cd is a hazardous element that is not beneficial to plants [61]. As indicated in Table 4, our results show that the concentrations of Cd in both the soil and plant tissues are rather low. The concentrations of Cd in plants range from 0.141 to 0.575 mg kg^−1^ DW in the aboveground parts and from 0.153 to 0.570 mg kg^−1^ DW in the root parts. These concentrations do not reach the Cd toxicity threshold, according to Krämer [50]. These low Cd concentrations can be attributed to the low levels of Cd found in the rhizosphere soils, as well as the limited bioavailability of this element. In Pb/Zn mining sites in northern Spain and northwestern Iran, native plant species accumulate the same concentrations of Cd ranging from 0.04 to 6.12 mg kg^−1^ DW in the aboveground parts and from 0.06 to 7.92 mg kg^−1^ DW in the roots (Barrutia, Artetxe [66]; Hosseinniaee, Jafari [67]). Midhat, Ouazzani [15] revealed Cd concentrations ranging from non-detectable to 0.37 mg kg^−1^ in the aerial parts and 15.99 mg kg^−1^ in the roots in several plants collected from Pb-Zn mining sites in the southern center of Morocco. In another study conducted by Hosseinniaee, Jafari [67], low concentrations of Cd ranging from 0.04 to 6.12 mg kg^−1^ in the aboveground parts and from 0.06 to 7.92 mg kg^−1^ in the roots were observed in plant tissues collected from a Pb-Zn site in northwest Iran. Among these plants, *Stipa arabica* and *Psathyrostachys fragilis*, *Astragalus verus*, *Centaurea virgata* and *Lactuca orientalis*, as well as *Chenopodium album*. The hyperaccumulation threshold for Cd is set at 100 mg kg^−1^ DW in the aboveground part of plants and is considered rare to achieve (Van der Ent, Baker [62]. This criterion categorizes none of the studied plants as Cd hyperaccumulators.

For Cd, the BAC values range from 0.363 for *Retama spherocarpa* to 1.38 for *Artemisia herba alba* (Table 5). With a BAC and TF greater than 1, *Artemisia herba alba* demonstrates the ability to translocate Cd to its aboveground part, which aligns with similar findings reported by El Hasnaoui et al. (2020) on Pb/Zn sites in eastern Morocco. Only three species recorded a TF less than 1, namely *Retama spherocarpa* (TF = 0.513), *Stipa tenacissima* (TF = 0.904) and *Astragalus armatus* (0.893). Only *Stipa tenacissima* showed a BCF greater than 1 (1.27), along with a BAC of 1.14 and TF of 0.904 for Cd, indicating the plant’s ability to accumulate Cd at the root level while limiting its translocation to the aboveground part. This suggests that *Stipa tenacissima* holds potential for Cd phytostabilization.

#### e. Antimony (Sb)

Antimony is not an essential element for plants [68,69]. The highest Sb concentration (1.15 mg kg^−1^ DW) was recorded in the aboveground part of *Astragalus armatus*, whereas the lowest concentration is 0.069 mg kg^−1^ DW in the root part of *Noaea mucronata*. The Sb concentrations found in all species do not reach the toxicity threshold as defined by Kabata-Pendias and Mukherjee [5] (Table 4). The low Sb concentrations accumulated by plants in general can be attributed to several factors, including the phytoavailability of Sb in soils, Sb uptake by plants, Sb speciation, and the levels of other ions in the soil, such as calcium (Ca) and phosphorus (P) [69,70]. Similar findings were reported by Hasnaoui et al. on two Pb/Zn sites in eastern Morocco, showing Sb concentrations in the root ranging from 1.6 mg kg^−1^ DW (*R*. *rigosum*) to 167.7 mg kg^−1^ DW (*S*. *tenacissima*), *while C*. *bursa-pastoris* and *A*. *herba-alba* accumulated the highest levels of Sb in their shoots (55.5 and 41.6 mg kg^−1^ DW, respectively). However, none of the species in our study reached the Sb hyperaccumulation threshold of 1000 mg kg^−1^ DW (Baker and Brooks [61].

None of the species studied exhibited a BAC and a BCF greater than 1 for Sb (Table 5). However, four species have a TF > 1 namely *Artemisia herba alba* (2.70), *Astragalus armatus* (4.35), *Salsola vermiculata* (1.35), and *Noaea mucronata* (2.34). This highlights the ability of these plants to translocate Sb to their aboveground parts. El Hasnaoui, Fahr [18] also observed a TF >1 for nine species collected from two Pb/Zn sites in eastern Morocco. These species include *R*. *alba* (3.55), *A*. *herba-alba* (3.2), *C*. *bursapastoris* (Touissite) (2.05), *S*. *hispanicus* L. (1.39), *A*. *alopecuroides* (1.19), *P*. *communis* (1.15), *H*. *incana* (1.2), and *C*. *libanotis* (1.13).

#### f. Zinc (Zn)

Zinc (Zn) is a crucial element for plant growth, readily taken up by roots from the soil, and then swiftly transported and stored in the aboveground parts in the form of free ions or simple organic acid complexes [15,71]. The concentrations of Zn in plants range from 8.69 mg kg^−1^ DW in the root part of *Noaea mucronata* to 263 mg kg^−1^ DW in the root part of *Astragalus armatus* (Table 4). *Astragalus armatus* accumulated the highest Zn concentration in its root part, falling within the toxicity range of 100 to 300 mg kg^−1^ DW as established by Kabata-Pendias and Mukherjee [5]. With the exception of the root part of *Astragalus armatus*, there are no significant differences in Zn concentrations among the studied species. In contrast to our study, Chehregani, Noori [72] found that *Noaea mucronata*, collected from Angouran Pb/Zn mine (Iran), is suitable for the phytoextraction of Zn. In a Pb/Zn site in northern Spain, Fernández, Poschenrieder [73] reported high concentrations of Zn in the aboveground parts of several species, including *Coincya monensis* (3391 mg kg^−1^), *Pteridium aquilinum* (L) Kuhn (2083 mg kg^−1^), *Minuartia verna* (3414 mg kg^−1^), *Silene ciliata* (1265 mg kg^−1^), and *Armeria cantabrica* Boiss & reut. Ex Willk. (1029 mg kg^−1^). The results of the present study showed that *Stipa tenacissima* recorded only 48.2 and 32.1 mg kg^-1^ DW in its aboveground and roots parts, respectively, whereas El Hasnaoui, Fahr [18] found a high concentration of Zn in the root part of *Stipa tenacissima* (637 mg kg^-1^ DW) collected from Pb/Zn sites in eastern Morocco. Midhat, Ouazzani [15] reported a concentration of Zn reaching 856.19 mg kg^−1^ DW in the roots of *Stipa capensis* Thunb, sampled from the mining site of Sidi Bou-Othmane, located in Marrakech, Morocco. However, none of the species in our study reached the Zn hyperaccumulation threshold of 10,000 mg kg^−1^ DW as reported by Baker and Brooks [61].

The BAC for Zn varied from 0.148 for *Noaea mucronata* to 0.776 for *Peganum harmala*, indicating that none of the studied species achieved a BAC ≥ 1 (Table 5). All species recorded a BCF < 1, except for *Astragalus armatus*, which showed a BCF of 2.26. This suggests that *Astragalus armatus* tends to accumulate Zn in its root part. Five species exhibited a TF ≥ 1, namely *Stipa tenacissima* (1.50), *Artemisia herba alba* (1.71), *Salsola vermiculata* (1.20), *Noaea mucronata* (1.48), and *Peganum harmala* (1.91). In contrast to our results, El Hasnaoui, Fahr [18] found that *Artemisia herba alba* is suitable for Zn phytostabilization (BCF > 1 and TF < 1). This can be explained by the variation in metal concentrations and bioavailability across the different sites, as well as the physicochemical characteristics of the soils, particularly the pH [53]. Only *Astragalus armatus* displayed a TF < 1 (0.263) and a BCF > 1 (2.26) for Zn, indicating its potential for Zn phytostabilization.

#### g. Lead (Pb)

Lead (Pb) is a non-essential and toxic element for plants [15]. Pb concentrations in plants range from 13.1 mg kg^−1^ DW in the root part of *Noaea mucronata* to 468 mg kg^−1^ DW in the aboveground part of *Artemisia herba alba*. *Stipa tenacissima* also accumulates a significant amount of Pb reaching 280 mg kg^−1^ DW in its root part (Table 4). With the exception of *Noaea mucronata*, all the other studied species recorded values exceeding the Pb toxicity threshold of 28 mg kg^−1^ DW as reported by Kabata-Pendias and Mukherjee [5], which means these species demonstrate a degree of tolerance to this metal. However, these values are lower than those found by El Hasnaoui, Fahr [18] in the mining site of Oued El Heimer, where Pb concentrations reached 3785.7 mg kg^−1^ DW in the root part of *Stipa tenacissima* and 4672.2 mg kg^−1^ DW in the aboveground part of *Artemisia herba alba*. Furthermore, Midhat, Ouazzani [15] reported an accumulation of 38.41 and 94.41 mg kg^−1^ DW respectively in the aboveground and root parts of *Citrullus vulgaris* grown in the Zn mining site of Bir Nehass site, in the southern center of Morocco. In the same study, they found a Pb concentration ranged from 121 and 282.33 mg kg^−1^ DW in aboveground part of *Forsskaolea tenacissima*, and *Stipa capensis* Thumb, respectively, [15]. In the present study, none of the studied species reached the Pb hyperaccumulation threshold of 1000 mg kg^−1^ DW. In contrast to our finding, several authors have reported that *Artemisia herba-alba* and *Stipa tenacissima*, growing on Pb/Zn mining sites, exceeded Pb hyperaccumulation threshold (1000 mg kg^−1^ DW) [18,74].

The BAC and BCF values for all species are less than 1 (Table 5). The BAC values for Pb varied from 0.009 for *Noaea mucronata* to 0.146 for *Artemisia herba alba*. The BCF values for Pb range from 0.007 for *Noaea mucronata* to 0.130 for *Stipa tenacissima*. *Artemisia herba alba* and *Salsola vermiculata* were the only species that exhibited a TF > 1, demonstrating their ability for Pb translocation. Despite the low BAC value, which can be related to the excessive Pb concentration in soil, these species may have potential for Pb phytoextraction.

#### h. Polymetallic accumulation capacity in plants

**Fig 4:**
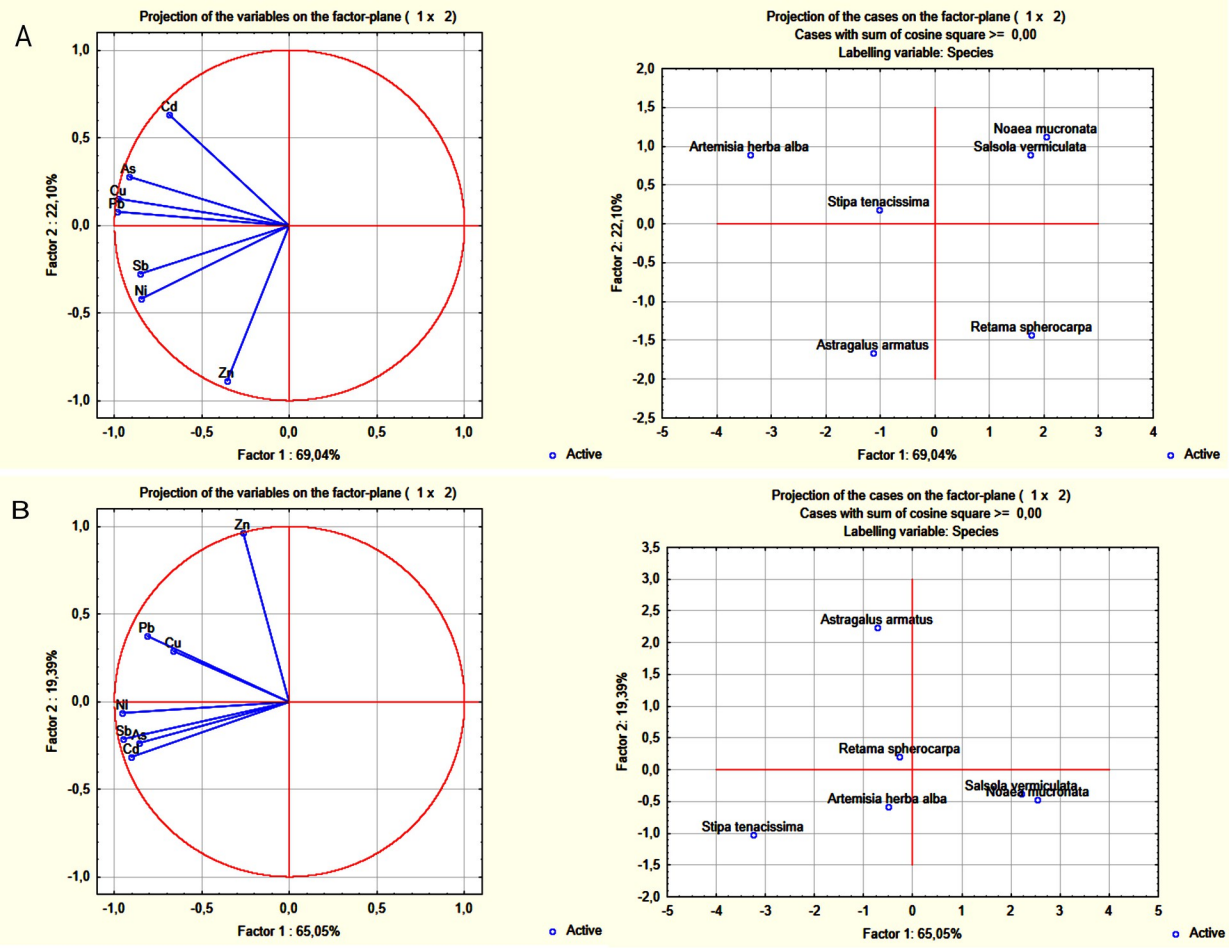
Principal Components Analysis (PCA) on the means of the metal(loid)s concentrations separately in the shoots (A) and the roots (B) for the studied species: *Artemisia herba alba*, *Stipa tenacissima*, *Astragalus armatus*, *Noaea mucronata*, *Salsola vermiculata*, and *Retama sphercarpa*.

Principal Component Analysis (PCA) was used to assess the polymetallic accumulation capacity in different parts of plant samples (aboveground and root parts) (Fig 4). The PCA biplot applied to metal concentrations in the aboveground parts of the studied plants demonstrates a total variance of 91.1% (Fig 4A). This variance, indicative of the plants ability to accumulate metal(loid)s, shows a positive correlation between *Artemisia herba alba* and Cd, As, Cu and Pb accumulation. This suggests the potential of this plant to concurrently accumulate multiple metal(loid)s in its aboveground part compared to other species (Fig 4A). These findings align with those of El Hasnaoui et al. (2020) who also demonstrated the high potential for polymetallic accumulation (As, Cd, Cu, Ni, and Pb) in the aboveground part of *Artemisia herba alba* harvested from Touissit Pb and Zn mining site. Du, Tian [75] also highlighted the ability of *Artemisia herba alba* to accumulate multiple metal(loid)s, including Pb, As and Hg. Additionally, *Astragalus armatus* exhibits a strong capacity to simultaneously accumulate Zn, Ni and Sb in its aboveground part (Fig 4A). Regarding the other species, *Noaea mucronata*, *Salsola vermiculata*, and *Retama spherocarpa* exhibit a negative correlation with polymetallic accumulation.

The PCA biplot applied to metal concentrations in the root parts reveals a total variance of 84.4% (Fig 4B). Both *Artemisia herba alba* and *Stipa tenacissima* exhibit a simultaneous accumulation capacity at the root level for Cd, As, Ni, and Sb (Fig 4B). Similar findings were reported by El Hasnaoui et al. (2020), demonstrating the strong and simultaneous accumulation capacity of *Artemisia herba alba and Stipa tenacissima* for As, Cd, Cu, Ni, Pb, Sb, and Zn in their root parts. Additionally, *Astragalus armatus* showed a positive correlation with Zn accumulation in roots.

## 5. Conclusion

Extended mining activities at the Zaida mining site in eastern Morocco have led to substantial heavy metal contamination, primarily attributed to poorly managed waste dumps. Soil analysis revealed elevated levels of heavy metal contaminants, notably Pb and Zn, with pollution index ranging from 1.6 to 10. Enrichment factor (EF) calculations corroborated these findings, indicating metal pollution levels exceeding natural background values. Furthermore, ecological risk assessment highlighted a significant potential risk index for Pb (Ei = 516.9).

While none of the plant species exhibited metal hyperaccumulation, *Artemisia herba alba* demonstrated notable accumulation capacities for Cd, As, Cu, and Pb in its aboveground parts. *Stipa tenacissima* exhibited promising potential for Ni and Cd phytostabilization, along with Zn phytoextraction, based on bioaccumulation factor (BAF), translocation factor (TF), and bioconcentration factor (BCF) analyses. *Astragalus armatus* also showed potential for Zn and Ni phytostabilization and demonstrated capabilities for As and Sb phytoextraction. These indigenous species from the abandoned Zaida Pb/Zn mining site could significantly contribute to metal(loid) phytoextraction and phytostabilization, as well as serve as vegetation cover to mitigate erosion and metal dispersion. However, further investigations are needed to elucidate the long-term effectiveness and scalability of these approaches in real-world remediation scenarios.

Considering the soil and climatic conditions of Zaida mining site, incorporating organic or biological amendments such as biochar, compost, or plant growth-promoting rhizobacteria (PGPR) is crucial. These amendments can enhance metal solubilization and immobilization, thus improving the efficacy of phytoremediation efforts [76]. Future investigations should focus on assessing the ecological impacts of phytoremediation, optimizing remediation strategies through the integration of multiple plant species and amendments, and evaluating the economic feasibility of implementing these techniques on a larger scale including the valorization of the harvested plant biomass in bioenergy production. Such endeavors will contribute to the development of sustainable and efficient remediation practices for contaminated sites like the Zaida mining area.

## Supporting information

S1 File (DOCX)
