# Supplementary material for: Native plant species growing on the abandoned Zaida lead/zinc mine site in Morocco: Phytoremediation potential for biomonitoring perspective
Source: PLoS One. 2024 Jun 26;19(6):e0305053. doi: 10.1371/journal.pone.0305053 (PMC11207124; doi:10.1371/journal.pone.0305053)
Supplement: S1 File — (DOCX) [file pone.0305053.s001.docx]

**Table 1: Coordinates of the various samples collected at the Pb/Zn site in Zaida**.

| Species | Repetition | Geographical coordinates |
| --- | --- | --- |
| *Retama spherocarpa* | 1 | 32° 50' 1.356'' N 4° 57' 7.308'' W |
|  | 2 | 32° 50' 5.064'' N 4° 57' 7.056'' W |
|  | 3 | 32° 50' 7.152'' N 4° 57' 8.844'' W |
|  | 4 | 32° 50' 5.496'' N 4° 57' 6.480'' W |
|  | 5 | 32° 50' 11.616'' N 4° 57' 16.74'' W |
| *Stipa tenacissima* | 1 | 32° 50' 11.688'' N 4° 57' 17.28'' W |
|  | 2 | 32° 50' 11.352'' N 4° 57' 15.636'' W |
|  | 3 | 32° 50' 8.988'' N 4° 57' 16.272'' W |
|  | 4 | 32° 50' 8.94'' N 4° 57' 17.952'' W |
|  | 5 | 32° 50' 8.976'' N 4° 57' 17.856'' W |
| *Artemisia herba alba* | 1 | 32° 50' 8.688'' N 4° 57' 17.1'' W |
|  | 2 | 32° 50' 8.736'' N 4° 57' 17.28'' W |
|  | 3 | 32° 50' 8.832'' N 4° 57' 17.496'' W |
|  | 4 | 32° 50' 8.868'' N 4° 57' 17.364'' W |
|  | 5 | 32° 50' 8.784'' N 4° 57' 17.28'' W |
| *Astragalus armatus* | 1 | 32° 50' 8.916'' N 4° 57' 16.944'' W |
|  | 2 | 32° 50' 8.82'' N 4° 57' 16.944'' W |
|  | 3 | 32° 50' 8.652'' N 4° 57' 16.944'' W |
|  | 4 | 32° 50' 8.616'' N 4° 57' 16.656'' W |
|  | 5 | 32° 50' 8.448'' N 4° 57' 17.244'' W |
| *Salsola vermiculata* | 1 | 32° 50' 9.768'' N 4° 57' 11.016'' W |
|  | 2 | 32° 50' 4.56'' N 4° 57' 6.084'' W |
|  | 3 | 32° 50' 4.632'' N 4° 57' 6.408'' W |
|  | 4 | 32° 50' 4.656'' N 4° 57' 6.384'' W |
|  | 5 | 32° 50' 4.656'' N 4° 57' 6.36'' W |
| *Noaea mucronata* | 1 | 32°47'10.518'' N 4°57'46.231'' W |
|  | 2 | 32° 47' 12.325'' N 4° 57' 45.133'' W |
|  | 3 | 32°47'11.936'' N 4°57'43.319'' W |
|  | 4 | 32° 47' 11.350'' N 4° 57' 44.089'' W |
|  | 5 | 32° 47' 10.377'' N 4° 57' 45.907'' W |
| *Peganum harmala* | 1 | 32°47'14.7"N 4°54'42.8"W |
|  | 2 | 32° 47' 14.9"N 4° 54' 42.8"W |
|  | 3 | 32° 47' 17.5"N 4° 57' 37.7"W |
|  | 4 | 32° 47' 14.5"N 4° 57' 42.7"W |
|  | 5 | 32° 47' 9.337'' N 4° 57' 45.753'' W |
